# Well-being of medical students and their awareness on substance misuse: a cross-sectional survey in Pakistan

**DOI:** 10.1186/1744-859X-8-8

**Published:** 2009-02-19

**Authors:** Abdul Wahab Yousafzai, Syed Ahmer, Ehsanullah Syed, Naila Bhutto, Saman Iqbal, Mohammed Naim Siddiqi, Mohammed Zaman

**Affiliations:** 1 Department of Psychiatry, Aga Khan University, Karachi 74800, Pakistan

## Abstract

**Objective:**

To investigate psychological well-being and substance abuse among medical students in Pakistan.

**Methods:**

A cross-sectional questionnaire-based survey was conducted in six medical colleges across Pakistan. Final-year medical students were interviewed by either a postgraduate trainee in psychiatry or a consultant psychiatrist.

**Results:**

A total of 540 medical students were approached; 342 participated and the response rate was 64.5%. Mean age was 23.73 years (SD 2.45 years); 52.5% were male and 90% single. Two out of every five respondents reported that work/study at medical school affected their personal health and well-being. A considerable proportion of students were aware of alcohol and smoking as coping strategies for stress in medical students. The main factors causing stress were heavy workload (47.4%), relationship with colleagues (13.5%) and staff (11.9%). A total of 30% reported a history of depression and 15% among them had used an antidepressant. More than half were aware of depression in colleagues. The majority of respondents said that teaching provided on substance misuse in the areas of alcohol and illegal drugs, management/treatment of addiction, and models of addiction was poor. There was significant association (p = 0.044) between stress and awareness about alcohol as a coping strategy for stress among medical students. A significant negative association was also found between medical colleges in public sector (p = 0.052), female gender (p = 0.003) and well-being.

**Conclusion:**

The majority of the medical students reported a negative impact of heavy workload on their psychological well-being. Significant numbers of medical students think that substance misuse is a coping strategy for stress. Teaching on addiction/addictive substances is poor at undergraduate level in Pakistani medical colleges.

## Introduction

Medicine has been a gratifying profession and held in high esteem since dawn of the history. It not only requires commitment, enthusiasm and altruism, but physicians are also expected to show care, compassion and a dedication to their profession.

Few studies have looked at stress related to medical education. Studies conducted in medical schools in the US and UK show a negative impact on student's physical and mental health [1-3]. A review of the literature by Liselotte *et al. *[4] showed the likely causes would be adjustments to the environment, ethical and moral dilemmas, exposure to human suffering, abuse, personal life events and debt. With passing years, the research highlights worsening distress and this can lead to impairment in academic performance, mental health problems and burnout.

A study from Newcastle, UK, showed that college students as a whole have a higher prevalence of alcohol drinking and alcohol use disorders than non-college youth [5]. Medical students therefore are a high-risk population. As high as 20% of first-year medical students admit to excessive alcohol intake, attributing it to stress, anxiety, and examination and work pressures [6]. A survey of eight US medical schools revealed 20% of students to have engaged in binge-drinking at least once in the past 30 days and 28% of students reported an increase in alcohol consumption during medical school [7].

Studies from Pakistan [8-10] have focused on perception of substance misuse, coping strategies and suicidal ideation among medical students. Our study looks at the well-being of medical students and their awareness on substance misuse, capturing data from six medical colleges across all the provinces of Pakistan.

## Materials and methods

Final-year medical students were approached in six medical colleges of four provinces in Pakistan to participate in the study. These include two from North-West Frontier Province (NWFP), two in Sind Province and one each in Baluchistan and Punjab.

In total 343 students participated in the survey.

We used a questionnaire which has already been used by British Medical Association (BMA) in survey of medical student's well-being in UK [11].

The questionnaire was in the English language, which is the medium of instruction in all medical colleges in Pakistan. The questionnaire consisted of seven items with multiple responses, covering psychological well-being of the students, their awareness about substance misuse, and depression. We collapsed some of the responses for the purpose of simplicity.

A demographic extraction sheet containing age, gender, marital status and background (whether rural or urban) was used to record the demographic variables. The data was collected by either a postgraduate trainee in psychiatry or consultant psychiatrist, and the anonymity of the respondent was insured. The study was approved by departmental ethical committee.

Data was entered in SPSS version 16.0 (SPSS, Chicago, IL, USA) and the frequencies of responses calculated. We also used the chi square test to assess the significance between different variables.

## Results

Out of a total of 540 medical students from the 6 medical colleges, 342 participated in the survey, yielding response rate of 64.5%. The mean age of respondents was 23.73 years (standard deviation (SD) 2.45 years), 52.5% were male and 90% were single.

With regard to ethnic distribution, 19.9% were Punjabis, 29.5% Pukhtuns, 11.5% Urdu speaking, 22.5% Sindis, and 7.6% Balochi. The number of students from rural and urban backgrounds was almost equal. The overwhelming majority (91%) were Muslims.

Tables 1 and 2 show the frequencies of responses. Three out of five respondents reported that work/study at medical school affected their personal health and well-being. Whilst 58.2% and 74.9% reported that they were not aware of smoking and alcohol use as a coping strategy for stress among medical students.

**Table 1:** Frequency of responses to stress-related questions

	**Responses**		
	
**Questions**	**Yes, n (%)**	**No, n (%)**	**Missing, n (%)**
Has your health been affected by stress?	234 (68.6)	96.1 (28.1)	11 (3.2)
Does work at medical school affect your personal health and well-being?	59 (17.3)	274 (82.7)	0
Are you aware of use of smoking in medical students to cope with stress?	134 (39.2)	199 (58.2)	7 (2.0)
Are you aware of use of alcohol in medical students to cope with stress?	78 (22.8)	256 (74.9)	6 (1.8)

**Table 2:** Frequency of responses to questions related to depression

**Questions**	**Responses**			
	
	**Yes, myself, n (%)**	**Yes, my colleagues, n (%)**	**Not aware, n (%)**	**Missing, n (%)**
Are you aware of depression amongst medical students?	103 (30.1)	178 (52.0)	52 (15.2)	9 (2.6)
Are you aware of use of antidepressants amongst medical students?	54 (15.8)	180 (52.6)	97 (28.4)	11 (3.2)

**Figure 1:**
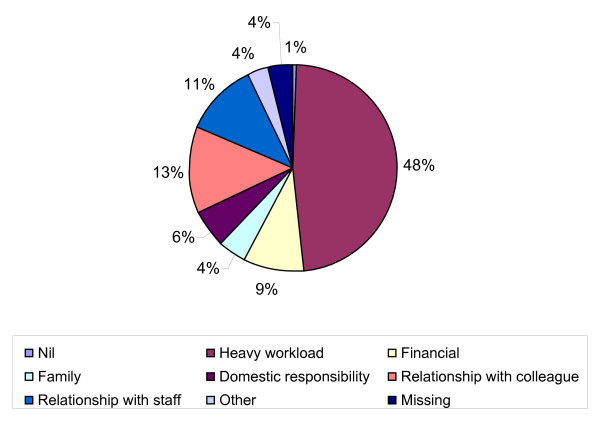
**Factors causing stress among medical students**.

Heavy workload (47.4%), followed by relationship with colleagues (13.5%) and staff (11.9%) appeared to be the main sources of stress as shown in figure 1.

While 30% of respondents reported that they have suffered from depression, only 15% have used antidepressant medications. More than half were aware of their colleagues suffering from depression and were using antidepressant medication.

The majority of the respondents were of the opinion teaching provided on substance misuse in the areas of alcohol, illegal drugs, management/treatment of addiction, and models of addiction is poor as shown in table 3.

**Table 3:** Feedback on teaching received on substance misuse

**Substances**	**Responses**		
	
	**Excellent, n (%)**	**Adequate, n (%)**	**Poor, n (%)**
Alcohol	67 (19.6)	106 (31.0)	163 (47.7)
Illegal drugs	48 (14.0)	98 (28.7)	190 (55.5)
Prescribed drugs	59 (17.3)	136 (39.8)	141 (41.0)
Over the counter drugs	30 (8.8)	103 (30.1)	203 (59.4)
Models of addiction	33 (9.6)	97 (28.4)	206 (60.2)
Management/treatment of addiction	29 (29)	94 (27.5)	213 (63.4)
Substance abuse by students/professionals	35 (10.2)	110 (32.2)	191 (55.8)
Prevention of substance abuse	36 (10.5)	93 (27.7)	207 (60.5)

Missing, 6 each (1.8).

The association between demographic variables and well-being is shown in Table 4. Government medical colleges (p value = 0.072), and female gender (p value = 0.003) had a significant association with stress. Alcohol use as coping strategy was found to be significantly associated with stress among medical students (p value = 0.044), as shown in Table 5.

**Table 4:** Demographic characteristics and association with well-being

**Variables**	**Does work at medical school affect your personal health and well-being?**		**p Value**
		
	**Yes**	**No**	
Type of college:			0.052
Private	43	10	
Government	165	75	
Gender:			0.003
Male	99	54	
Female	113	28	

Values statistically significant at the level of p < 0.05.

**Table 5:** Demographics and alcohol use in association with stress

**Variables**	**Has your health ever been affected by stress?**		**p Value**
		
	**Yes**	**No**	
Type of college:			0.164
Private	48	6	
Government	195	46	
Gender:			0.626
Male	130	27	
Female	118	21	
Awareness of alcohol use to cope with stress:			0.044
Yes	70	8	
No	201	51	

Values statistically significant at the level of p < 0.05.

## Discussion

This study is part of a larger project covering well-being and bullying of medical students in Pakistan. Other findings will be reported separately.

The training period for medical students is a constantly changing environment of 5 to 6 years, to ensure that graduates gain sufficient skills. Some aspects of the training have been found to have negative effects on the student's life, which manifest in the form of stress, depression and burn out.

An important finding of this survey is the prevalence of stress among medical students; about 65% of them found the training period stressful. This finding is in agreement with other studies conducted in Pakistan [8] and elsewhere [4].

An alarming result of this study is the awareness of a significant number (39.2%) of students about smoking and alcohol (22.8%) as coping mechanisms for stress in their colleagues. This finding lends support to previous research from Pakistan, showing that 90% of medical students perceived academic stress as being responsible for drug use among medical students [8]. Drug use among medical students is a worldwide phenomenon; for example, a survey of an American medical school showed alcohol, benzodiazepine and opiate use to be higher than age-matched controls [12]. Moreover, alcohol and drug addiction represents 80% to 90% of all physician impairment cases in the US, and is a source of major concern for health authorities [13].

With regard to the main factors responsible for causing stress, the majority of the students attribute it to heavy workload (47.4%) followed by relationship with colleagues (13.5%) and staff (11.9%). This finding is consistent with a BMA survey in the UK [11]. Similarly, workload such as academic studies and exams have been found to be major sources of stress among Pakistani medical students [10] though such workload varies for medical students on a yearly basis and is, usually, coupled with concerns for academic performance [4].

Almost 40% (40.1%) of participants reported a history of depression, while more than 50% were aware of depression among their fellow students. This is the most striking finding of our study. Similar findings have been reported in other research from Pakistan; for example, one study reported suicidal ideation in a third of Pakistani medical students [9]. In part this could be a reflection of a high prevalence of common mental disorders in Pakistan [14].

Various studies from US have identified a high frequency of depression and suicidal tendencies among medical students; in fact, suicide ranks second among the leading causes of death in medical students [15]. In a UK survey, about 14% of medical students reported depression while 53% were found aware of depression amongst their colleagues.

The medical students in this survey reported poor teaching on substance abuse. This could be explained by the fact that psychiatry is not taught as a major subject at undergraduate level in Pakistan except in a few medical colleges. As such it is understandable that students will be less likely to know a great deal about substance abuse problems.

Female medical students and government-run institutions have been found to be negatively associated with health and well-being of medical students. This finding is consistent with studies conducted previously in Pakistan [10] and elsewhere [16]. Regarding government institutes, generally less institutional support is available to the students in Pakistan. Another reason could be the fact that less affluent students only gain admission into public institutions, which could make them more vulnerable to stress.

A significant association between stress and awareness of alcohol use as a coping strategy for stress is an important finding of this study. This finding necessitates the need for further research to study actual alcohol use in stressed medical students.

### Limitations

This is a preliminary study concerning medical students and substance use, with obvious limitations. Direct figures were not obtained on actual patterns of substance misuse among medical students, and associations were observed. Additionally, no attempts were made to interview students for current depressive symptoms. For psychological well-being, self-report was used instead of standard scale measurements.

Our sample may also not be representative of the population; for example, only one medical college was selected from Punjab, which is the largest province of Pakistan.

## Conclusion

High levels of distress have been reported by medical students in Pakistan, and a significant proportion reported that their well-being has been affected by stress. The vast majority of medical students reported that they know of colleagues who use alcohol and smoking to cope with stress. Moreover, workload was cited by the majority of students as the source of stress.

Depression among medical students is high, as reported by the students in this survey. There is general agreement amongst medical students that the teaching of substance abuse in medical schools is over all poor.

## Competing interests

The authors declare that they have no competing interests.

## Authors' contributions

AWY Conceived the idea and also took part in collection and analysis of data and wrote the initial draft of the article. SA took part in data analysis and manuscript writing. ES: did literature search and reviewed the manuscript while NB collected the data and retrieved the relevant references. SI: Analyze the data, and reviewed the manuscript. MNS: Reviewed the manuscript. MZ: entered and analyzed the data.
